# Heterogeneity of *O*^6^-alkylguanine DNA-alkyltransferase expression in human breast tumours

**DOI:** 10.1038/sj.bjc.6600324

**Published:** 2002-06-05

**Authors:** M J Clemons, M C Bibby, H El Teraifi, G Forster, J Kelly, S Banerjee, B Cadman, W D J Ryder, A Howell, G P Margison

**Affiliations:** Cancer Research UK Department of Medical Oncology, Christie Hospital, Wilmslow Road, Manchester M20 4BX, UK; Cancer Research UK Carcinogenesis Group, Paterson Institute for Cancer Research, Christie Hospital, Wilmslow Road, Manchester M20 4BX, UK; Cancer Research Unit, University of Bradford, Bradford BD7 1DP, UK; Department of Cytology, Christie Hospital, Wilmslow Road, Manchester M20 4BX, UK; Department of Pathology, Christie Hospital, Wilmslow Road, Manchester M20 4BX, UK; Department of Pathology, Bradford Royal Infirmary, Bradford BD7 1DP, UK; Department of Statistics, Christie Hospital, Wilmslow Road, Manchester M20 4BX, UK

**Keywords:** DNA repair, prognostic indicators, MGMT, nitrosoureas, methylating agents

## Abstract

An important determinant of cellular resistance to chemotherapeutic *O*^6^-alkylating agents, which comprise methylating and chloroethylating agents, is the ability of cells to repair alkylation damage at the *O*^6^-position of guanine in DNA. This is achieved by a specific DNA repair enzyme *O*^6^-alkylguanine DNA-alkyltransferase. In this study *O*^6^-alkylguanine DNA-alkyltransferase expression was measured in human breast tumours using both biochemical and immunohistochemical techniques. *O*^6^-alkylguanine DNA-alkyltransferase activity was then compared with known clinical prognostic indices to assess the potential role of *O*^6^-alkylguanine DNA-alkyltransferase in predicting the behaviour of this common malignancy. The application of both biochemical and immunohistochemical techniques was feasible and practical. Most breast tumours expressed high levels of *O*^6^-alkylguanine DNA-alkyltransferase. Immunohistochemical analysis showed marked variation in expression not only between individuals but also within individual tumours, and in the same patient, between metastases and between primary tumour and metastatic site. *O*^6^-alkylguanine DNA-alkyltransferase activity in tissue extracts significantly correlated not only with immunohistochemical staining intensity determined by subjective quantitation, but also with measures of protein levels using a computerised image analysis system including mean grey (*P*<0.001), percentage of cells positive for *O*^6^-alkylguanine DNA-alkyltransferase (*P*<0.001), and integrated optical density (*P*<0.001). *O*^6^-alkylguanine DNA-alkyltransferase expression did not correlate with any of the established clinical prognostic indicators for current treatment regimens. However, immunohistochemical offers a rapid and convenient method for assessing potential utility of *O*^6^-alkylating agents or *O*^6^-alkylguanine DNA-alkyltransferase inactivating agents in future studies of breast cancer treatment.

*British Journal of Cancer* (2002) **86**, 1797–1802. doi:10.1038/sj.bjc.6600324 www.bjcancer.com

© 2002 Cancer Research UK

## 

Standard first-line chemotherapy for advanced breast cancer results in disease regression in the majority of patients but these responses are rarely complete or sustained (Clemons *et al*, 1997). Patients with relapsed or persistent disease often require further chemotherapy and there is, therefore, a continuing need to develop new treatments. One strategy is the biochemical modulation of tumour drug resistance, an important area of experimental and clinical cancer therapeutics.

The chemotherapeutic *O*^6^-alkylating agents are well established in oncology and are a component of high-dose therapy regimens currently used in the treatment of metastatic (Peters *et al*, 1994; Antman, 2001), and high-risk adjuvant breast cancer (Bengala *et al*, 2001). These agents include methylating (e.g. Dacarbazine and Procarbazine) and several chloroethylating agents (e.g. Carmustine and Semustine).

Both *in vitro* and *in vivo* experiments have demonstrated that an important determinant of resistance to *O*^6^-alkylating agents is the ability of cells to repair alkylation damage at the *O*^6^-position of guanine in DNA. This is achieved by the DNA repair protein, *O*^6^-alkylguanine DNA-alkyltransferase (ATase), through the covalent transfer of the alkyl group from the *O*^6^ position of guanine to a cysteine residue within the active site of the ATase protein, a reaction that is stoichiometric and autoinactivating (Pegg, 1990, 1994; Pegg *et al*, 1995).

The relationship between the sensitivity of human tumours *in vivo* and ATase levels is complex (D'Incalci *et al*, 1998; Phillips and Gerson, 1999) and has as yet not been fully elucidated, although pre-clinical studies have demonstrated a correlation between tumour ATase levels and sensitivity to nitrosoureas (Margison and O'Connor, 1990; Pegg, 1990). Several authors have measured ATase expression in human breast tumours and report low (Cao *et al*, 1991), moderate (Chen *et al*, 1992) and high activity (Musarrat *et al*, 1995). Considerable heterogeneity of ATase levels is also reported for other tumour types (Gander *et al*, 1999). High levels of ATase activity have been correlated with a poor prognosis in early breast cancer (Citron *et al*, 1994) and may in part explain the relative resistance of metastatic breast cancer to treatment with *O*^6^-alkylating agents. A number of strategies are currently under clinical trial in breast cancer patents using either alkylating agents (NCIC, 2001) or pseudosubstrates (Spiro *et al*, 1999) to deplete tumour ATase and hence increase tumour chemosensitivity. Indeed recent work has used changes in tumour ATase expression for calculating the effective dose of ATase modulators in Phase I studies (Spiro *et al*, 1999).

In view of these ongoing strategies for ATase depletion it is important to have useful measures of tumour ATase activity. Conventional assays of ATase expression in extracts of biopsy material are extremely sensitive but produce tissue-average values and take no account of cellular heterogeneity (Lee *et al*, 1991; Watson and Margison, 2000). In order to assess the inter- and intra-cellular distribution of the protein a sensitive and highly specific antiserum to recombinant human ATase (Lee *et al*, 1992) was used in formalin-fixed paraffin embedded breast tumours. Having shown the technique to be feasible ATase expression was measured in snap-frozen breast tumours by both conventional biochemical and by immunohistochemical (IHC) assay. IHC analyses of ATase expression scored both subjectively and objectively with a computerised quantitative digital IHC technique were then compared. The computerised system should be less subjective, more quantitative and potentially more sensitive than conventional human visualisation methods. Indeed, computerised scoring is already established for the analysis of oestrogen and progesterone receptor status in human breast tumours (Van Agthoven *et al*, 1995).

Finally, statistical analyses were performed to determine if ATase expression measured by assay or IHC were correlated either with each other or with other clinical prognostic parameters present in these patients. The mechanisms of regulation of expression of ATase are poorly understood and whilst there is no *a priori* basis for comparing prognostic variables with ATase, the possibility of a correlation between it and other variables was considered a worthwhile question.

## MATERIALS AND METHODS

### Samples

#### Formalin-fixed samples from primary breast tumours

Tissue specimens from patients undergoing surgical treatment for primary breast cancer at Bradford Royal Infirmary were fixed in buffered formalin and embedded in wax. Sections stained with haematoxylin and eosin (H&E) were used for routine pathological diagnosis while other sections were stained for ATase (see below).

#### Alcohol-fixed samples from primary breast tumours

Samples for resected tumours obtained at the time of primary surgery at the Christie Hospital were snap-frozen in liquid nitrogen and stored until subsequent assay. Samples were rapidly defrosted in batches of 10 and each tumour divided into two parts. Half was immediately assayed for ATase activity and the other half fixed in 70% alcohol for subsequent IHC. These sections were assessed for ATase using a conventional IHC scoring method and an image analysis system. Clinical, staging and prognostic information was available for all these patients.

### Measurement of ATase activity

The preparation of tissue extracts and quantitation of ATase activity was performed as previously described (Watson and Margison, 2000).

### IHC staining and quantitation

Sections (3 μm) were prepared, mounted onto 3-aminopropyltriethoxysilane (Sigma Chemical Co. Ltd., Poole, UK) coated slides, dried overnight, dewaxed in xylene and rehydrated through graded alcohols (100%, 90%, 70% and 40% ethanol). Sections of formalin fixed material were microwaved for 25 min in 1 l of 0.1 M citric acid pH 6.0 at this stage to retrieve antigenic sites. After washing in 50 mM TBS (pH 7.6) endogenous peroxidase activity was blocked with 3% H_2_O_2_ in Tris-buffered saline (TBS) for 15 min at room temperature and washing in running water followed by TBS. The sections were incubated with 5% normal swine serum in TBS for 20 min at room temperature to block nonspecific binding of IgG, and were then incubated with polyclonal rabbit anti-human ATase antiserum (1 : 1500) in TBS overnight at 4°C. Control sections were incubated with pre-adsorbed serum at the same dilution. After washing in TBS the slides were incubated with biotinylated swine anti-rabbit serum at 1 : 400 dilution for 30 min at room temperature, washed and then incubated with an avidin-biotin-horseradish peroxidase complex (ABC, Vector Laboratories Ltd., Peterborough, UK) for 45 min. Sections were finally developed with 3,3-diaminobenzidine (DAB/H_2_O_2_) for 5 min, washed well in TBS and counterstained with the red fluorescent DNA dye propidium iodide (5 μg ml^−1^ in PBS for 30 min at room temperature).

### Conventional IHC scoring

Each specimen was assessed by a consultant histopathologist (HE-T, BC) and for each biopsy, 100–400 tumour cells were counted and the intensity of staining of each cell determined visually and classified semi-quantitatively: negative (0)=no staining; 1+=weak staining; 2+=medium staining; 3+=strong staining, 4+=very strong staining. The fraction of negative, 1+, 2+, 3+ and 4+ cells was calculated for each tumour and staining patterns discriminated as nuclear, cytoplasmic or both.

### Image analysis

The intensity of ATase staining was measured using a Lucia Image Analyser (Nikon UK Ltd., Kingston upon Thames, UK). Duplicate sections were prepared for each tumour, one section was stained with primary antibody, and the other with antigen adsorbed serum (control). Threshold levels were set using the staining of the control section as a cut off point for each patient. This did not vary by greater or less than three grey levels within the 255 levels of the dynamic range of the system. Any staining above this threshold was counted as positive staining. Ten fields were counted using the ×20 objective lens and for each field several parameters were analysed (see below), the total area of the DAB stained cells measured and the binary image recorded. Fluorescent green light (excitation 540/24; dichroic mirror 565; barrier 605/55) was used to highlight the propidium iodide present in ATase negative cell nuclei in the field and these were thresholded. The ATase positive binary image was added to the propidium iodide positive binary image and the total nuclear area recorded. This allowed expression of all image analysis parameters as a function of total nuclear area in the subsequent quantitative assessment. The intensity of the labelling was determined by the computer programme and gave a grey value from 0 (black) to 255 (white). After capturing the image on the computer screen, stained areas were identified as those regions with a grey value for each pixel below the threshold, which was set at 230.

The parameters assessed in the detected areas were: (1) Mean grey: the sum of all the grey levels in the tumour cells divided by the number of positively staining cells. This was averaged over all the fields viewed. (2) Per cent positivity: the number of cells positively staining for ATase (using the pre-adsorbed control field as the threshold) as a percentage of the total number of cells. (3) Integrated optical density (IOD): the sum of grey value for each pixel in the detected area, indicating the total amount of light-absorbing material in the defined region. (4) Integrated optical density per area: IOD divided by the total area covered by breast cancer cells. This value was compared with the level of ATase (fmoles mg^−1^ of protein) measured by biochemical assay of tissue extracts. (5) The percentage of cells displaying a grey value of <100. This gives an estimate of the distribution of highly stained cells in a sample: the grey value was arbitrarily selected.

### Statistical analysis

For the alcohol-fixed series three methods of tumour ATase quantitation were used; biochemical assay (quantified with respect to cellular protein and DNA content), IHC semi-quantitative scoring by a histopathologist and IHC followed by computerised image analysis. Each of these results were compared with a series of established prognostic variables available for that particular patient. This prognostic information included tumour size, grade, histological type, Manchester score (this combines the node ratio (the percentage of axillary lymph nodes positive for disease divided by the total number of lymph nodes removed), menopausal status, tumour grade and whether or not axillary lymph nodes are clinically positive), axillary lymph node status as well as tumour oestrogen receptor (ER), progesterone receptor (PR), Ki 67 and epidermal growth factor receptor (EGFR) expression.

Due to the large number of variables being assessed, simple correlations were used. Spearman's rank pair correlation was used to compare the individual data points in ordered rank pairs. The Kruskal–Wallis one-way ANOVA test was used to compare differences in the median score between particular groups. For those sets of data where only two groupings existed (e.g. EGFR: absent or present) the Mann–Whitney *U*-test was used. For the comparisons with the subjective assessment of ATase expression, rank correlations were again used as the score (0, 1+, 2+, 3+, 4+) is naturally ordered.

## RESULTS

### Formalin-fixed samples from primary breast tumours

Sixty-one primary breast tumours were analysed. Fifty-five of the tumours were ductal carcinomas, six lobular carcinomas, one spindle cell and one medullary carcinoma. Of the ductal carcinomas 12 were histopathological Grade 1, 24 were Grade 2 and 17 were Grade 3 (Elston, 1984) (Table 1

**Table 1:**
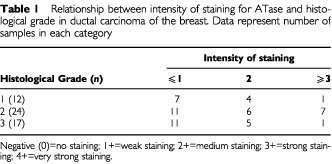
Relationship between intensity of staining for ATase and histological grade in ductal carcinoma of the breast. Data represent number of samples in each category

).

ATase staining was seen in most of the tissues studied, only in two tumours was ATase undetectable. In both tumour and normal breast, ATase was detected predominantly in the nuclei, however cytoplasmic expression was also seen. ATase staining varied markedly from one patient to another. There was also heterogeneity within tumours; in some tumours, most of the cells expressed the protein in a relatively homogenous pattern, whereas in others, only a few of the cells were stained either as isolated regions within the tumours or as isolated cells.

**Figure 1:**
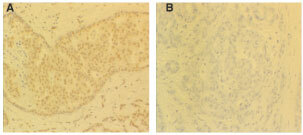
Photomicrographs of IHC staining for ATase in formalin-fixed breast tumour (×200). (**A**) Heterogeneous, high level (3+) predominantly nuclear staining with some weak cytoplasmic staining (+) in both tumour and surrounding parenchymal tissues. (**B**) Pre-adsorbed control.

While staining was mainly confined to the nuclei it was not restricted to tumour cells. In many cases intense staining was seen in nuclei of endothelial cells and fibroblasts, normal duct epithelial cell nuclei as well as nuclei of infiltrating carcinoma cells (Figure 1

**Figure 3:**
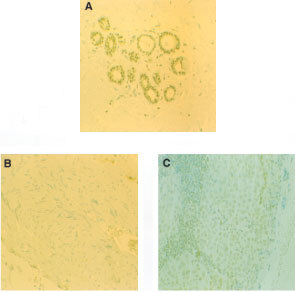
Photomicrographs of IHC staining for ATase in different breast tumours from the same patient (×200). (**A**) High ATase staining (3+) in normal breast; (**B**) no staining (−) in primary breast tumour; (**C**) variable staining in a metastatically infiltrated axillary lymph node. Also shows capsular infiltration by tumour.

). Tumours sometimes had marked lymphocyte infiltration and in these cases, there was intense staining in many lymphocyte nuclei.

There was no clear pattern of staining intensity that could be related to histological grade (Table 1). Of the five lobular carcinomas scored, four had high intensity staining and one had moderate intensity. The single case of spindle cell carcinosarcoma examined demonstrated high intensity nuclear staining.

### Alcohol-fixed samples from primary breast tumours

Sixty-three tumours were analysed and the patient tumour and staging characteristics are shown (Table 2

**Table 2:**
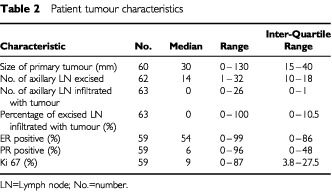
Patient tumour characteristics

). Using the biochemical assay, ATase activity was detected in 62 out of 63 of the breast tumour samples studied. The median and ranges of ATase activity by the different methods of assessment are shown (Table 3

**Table 3:**
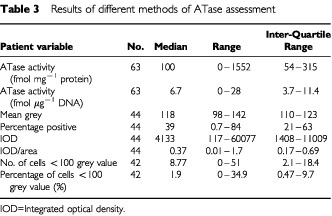
Results of different methods of ATase assessment

). Figure 2

**Figure 2:**
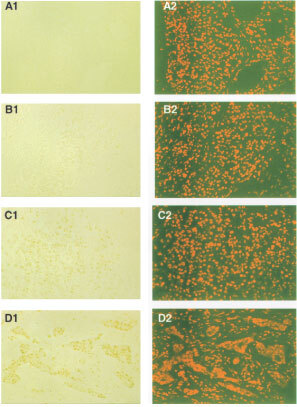
Photomicrographs of IHC staining for ATase in alcohol fixed breast tumours (×200). (**A1**) pre-immune serum control (−); (**B1**) low (1+); (**C1**) medium (2+) and (**D1**) high (3+) staining. A2-D2 show cells negative for ATase stained with propidium iodide (red fluorescence).

shows examples of the five subjective grades for ATase IHC stain intensity, along with their controls.

Known prognostic variables were compared with the various measures of ATase activity using the Kruskal–Wallis one-way ANOVA. Table 4

**Table 4:**
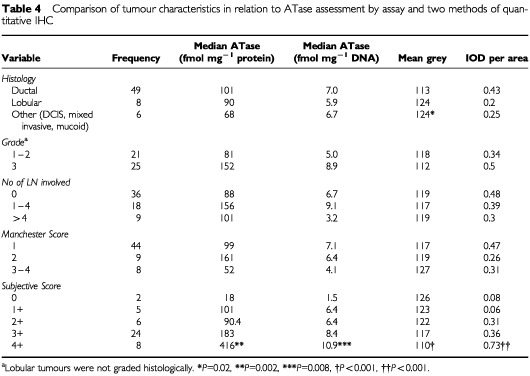
Comparison of tumour characteristics in relation to ATase assessment by assay and two methods of quantitative IHC

shows the patient tumour and staging characteristics in relation to ATase expression by assay and by two methods of quantified IHC. As many of the results were positively skewed the median values and ranges are given, and log values used to normalise the data for the statistical analyses. Because two tumours lacked any ATase activity, the log (*x*+1) was taken, where *x* is the *in vitro* assay result used in the correlation. Patient information for all of the variables was not always available hence, the number of data points available for each variable was not always the same.

Data for 63 patients was available and of these, 54 were alive with no evidence of disease recurrence at the time of data analysis, three had relapsed and six had died. Insufficient actual survival data was therefore available at this stage to assess whether or not any of the different methods of measuring ATase activity were correlated with the patient's ultimate prognosis. No measure of activity was correlated with lymph node status, tumour grade and Manchester Score.

While it appeared that ductal tumours had greater intensity of staining (i.e. lower mean grey) only a relatively small number of samples with non-ductal tumours were analysed. The subjective IHC staining intensity score was significantly correlated not only with the ATase activity by protein (*P*=0.002) and DNA (*P*=0.008) but also with mean grey (*P*<0.001), percentage of cells positive for ATase (*P*<0.001), IOD (*P*<0.001), IOD/area (*P*<0.001) and number (%) of cells <100 grey (*P*<0.001). (Table 4)

As most studies cite ATase expression in human tumours with respect to either DNA or protein content, these two methods of ATase assessment were compared with each other and were found to be significantly correlated with each other (*P*<0.001).

## DISCUSSION

The chemotherapeutic *O*^6^-alkylating agents are well established in oncology and are a component of high-dose therapy regimens currently used in the treatment of metastatic (Peters *et al*, 1994; Antman, 2001), and high-risk adjuvant breast cancer (Bengala *et al*, 2001). Since ATase is the principal mechanism of cellular resistance to *O*^6^-alkylating agents, its role in tumour aetiology and response is of considerable interest. Other studies have found that while ATase is expressed by all breast cell types (Chen *et al*, 1992), particularly high levels have been seen in breast cancer cells (Musarrat *et al*, 1995). This has been proposed to explain in part the relative resistance of breast cancer to treatment with *O*^6^-alkylating agents. In Citron's study (Citron *et al*, 1994) while 41% of normal breast tissue was ATase deficient only 4% of malignant tissue was. ATase levels were also positively correlated with the number of infiltrated axillary lymph nodes supporting the hypothesis that ATase activity increases with disease progression.

ATase activity has previously been measured in tissue extracts and this obscures intercellular heterogeneity. This might be an important issue in the response of individual cells to treatment. In this present study we have investigated the distribution of ATase in fixed tissues in relation to biochemical measurements of ATase activity in tumour tissue extracts and in relation to several clinical parameters.

Utilisation of both biochemical and IHC techniques was feasible and practical. Inspection of the histological sections from the formalin-fixed breast tumours revealed that staining was highly heterogeneous, from cells completely devoid of ATase to cells with very intense staining. Marked variation in expression was also seen between individuals. Many patients demonstrated high staining intensity in nuclei suggesting high levels of repair protein but in our study there was no clear evidence that malignant cell nuclei contained higher levels of protein compared to normal cells within the same section (e.g. in fibroblasts, endothelial, duct cells and neutrophils).

The median ATase activity of the 63 tumour samples was 100 fmol mg^−1^ protein and 6.7 fmol μg^−1^ DNA which is consistent with previously reported series (Cao *et al*, 1991; Chen *et al*, 1992; Musarrat *et al*, 1995). There were significant correlations between ATase activity by assay expressed as fmol μg^−1^ DNA and fmol mg^−1^ protein, and between the activity expressed per mg protein with the mean grey, percentage positive and IOD scores. It was not surprising that many of the quantified IHC scores were correlated with each other as many of the values used in their calculation were common to all of them. It was also of note that the subjective assessment of the intensity of ATase expression by IHC was significantly correlated with many of the assay and image analysis systems scores. This provides further evidence for the usefulness of subjective IHC but also adds further validity to the use of the computerised system: currently, subjective assessment alone is used in many areas of clinical practice.

The breast tissue series that were alcohol-fixed were assessed by both biochemical and IHC techniques. The estimates were then compared with a series of known prognostic variables. The purpose of this was not solely to establish if there were correlations of statistical significance *per se* but also to define which of these parameters might prove useful in future larger multiparametric studies. However, none of the other variables, including tumour size, grade, histological type, Manchester score, axillary lymph node status, tumour oestrogen receptor, progesterone receptor, Ki 67 and epidermal growth factor receptor expression, correlated with ATase. Most patients had early, grade 2 ductal carcinomas and this too made correlation between ATase activity and tumour stage/grade/histological type less accurate.

There have been relatively few similar studies on which to base an assessment of whether biochemical assay or IHC is the clinically relevant score. Zaidi *et al* (1996) compared ATase activity in human colon tissue by assay, Western blotting and conventional IHC. They also used a digital image analysis system to quantify ATase activity and correlated these results with those of the more ‘conventional’ techniques. They found that ATase estimates by biochemical and Western blots were strongly correlated (*P*<0.0001). However, the Western blots were quantitated on the basis of control lanes loaded according to ATase activity and not total ATase protein and this might not be ideal. Two parameters of quantitative IHC, integrated grey and mean grey, were also correlated with each other (*P*<0.002) and with biochemical and Western blot estimates (*P*=0.004–0.04).

**Figure 4:**
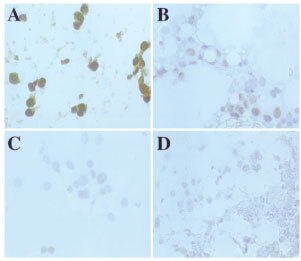
Photomicrographs of IHC staining for ATase in alcohol fixed breast tumours (×200). Variation in IHC staining for ATase in four separate breast tumour sites from the same patient. (**A**) very high (4+); (**B**) medium (2+); (**C**) low (1+) and (**D**) no staining (−) intensity. In all cases the staining is mainly nuclear in distribution.

The present study indicates that there is very extensive heterogeneity of ATase staining and that separate biopsies of the same primary tumour (Figure 4

) or biopsies from individual metastases would not represent ATase levels in the disease as a whole. These IHC results are similar to those previously seen in metastatic malignant melanoma where marked variation in ATase staining was seen among tumour cells within primary tumours, within the same metastasis, and between separate metastases in the same patient (Lee *et al*, 1993; Egyhazi *et al*, 1997). This heterogeneity of staining for ATase has been assumed to influence the variation of response of metastatic melanomas to chemotherapeutic *O*^6^-alkylating agents such as DTIC (Egyhazi *et al*, 1997). Heterogeneity might also explain the absence of predictive potential of measuring ATase activity levels in biopsy homogenates in a Phase II study of melanoma patients treated with temozolomide (Middleton *et al*, 1998). It is highly likely that patients whose tumours express low ATase activity would obtain a better response with *O*^6^-alkylating agents than those with tumours expressing higher levels. However, given the heterogeneity of ATase expression, individual, or even multiple, biopsies and IHC assessment or *in vitro* assay is unlikely to be a consistent predictive parameter of response.

## References

[bib1] AntmanKH2001 Randomized trials of high dose chemotherapy for breast cancer Biochim Biophys Acta 1471 8998 10.1016/s0304-419x(00)00023-8 11250065

[bib2] BengalaCPazzagliIInnocentiFDonatiSFavreCMenconiMCGrecoFDanesiROrlandiniCGuarneriVDel TaccaMContePF2001 High-dose thiotepa and melphalan with hemopoietic progenitor support following induction therapy with epirubicin-paclitaxel-containing regimens in metastatic breast cancer (MBC) Ann Oncol 12 6974 11249051 10.1023/a:1008302402687

[bib3] CaoE-HFanXJYuanXHXinSMLiuYYYuHT1991 Levels of *O*^6^-methylguanine acceptor protein in extracts of human breast tumour tissues Cancer Biochem Biophys 121 5358 1769005

[bib4] ChenJMZhangYPWangCSunYFujimotoJIkenegaM1992 O6-Methylguanine-DNA Methyltransferase Activity in Human Tumours Carcinogenesis 13 15031507 1394831 10.1093/carcin/13.9.1503

[bib5] CitronMSchoenhausMRothenbergHKostroffKWassermanPKahnLWhiteABurnsGHeldDYaroshD1994 O6-methylguanine-DNA methyltransferase in normal and malignant-tissue of the breast Cancer Investigation 12 605610 7994595 10.3109/07357909409023045

[bib6] ClemonsMJLeahyMGValleJJaysonGRansonMHayesSHowellA1997 Review of recent trials for advanced breast cancer. Part I: Studies excluding taxanes Eur J Cancer 33 21712182 9470803 10.1016/s0959-8049(97)00262-1

[bib7] D'IncalciMBonfantiMPifferiAMascellaniETagliabueGBergerDFiebigHH1998 The antitumour activity of alkylating agents is not correlated with the levels of glutathione, glutathione transferase and *O*^6^-alkylguanine-DNA-alkyltransferase of human tumour xenografts. EORTC SPG and PAMM Groups Eur J Cancer 34 17491755 9893664 10.1016/s0959-8049(98)00191-9

[bib8] EgyhaziSMargisonGPHanssonJRingborgU1997 Immunohistochemical Examination of the Expression of *O*^6^-methylguanine-DNA Methyltransferase in Human Melanoma Metastases Eur J Cancer 33 129134 9071912 10.1016/s0959-8049(96)00342-5

[bib9] ElstonCW1984 The assessment of histological differentiation in breast cancer Aust N Z J Surg 54 1115 6586161 10.1111/j.1445-2197.1984.tb06677.x

[bib10] GanderMLeyvrazSDecosterdLBonfantiMMarzoliniCShenFLienardDPereyLColellaGBiollazJLejeuneFYaroshDBelanichMD'IncalciM1999 Sequential administration of temozolomide and fotemustine: depletion of O6-alkyl guanine-DNA transferase in blood lymphocytes and in tumours Ann Oncol 10 831838 10470431 10.1023/a:1008304032421

[bib11] LeeSMThatcherNMargisonGP1991 *O*^6^-alkylguanine-DNA alkyltransferase Depletion and Regeneration in Human Peripheral Lymphocytes Following Dacarbazine and Fotemustine Cancer Res 51 619623 1824685

[bib12] LeeSMRaffertyJAElderRHFanC-YBromleyMHarrisMThatcherNPotterPMAltermattHJPerinat-FreyTCernyTO'ConnorPJMargisonGP1992 Immunohistological examination of the inter- and intracellular distribution of *O*^6^-alkylguanine DNA-alkyltransferase in human liver and melanoma Br J Cancer 66 355360 1503911 10.1038/bjc.1992.270 PMC1977793

[bib13] LeeSMHarrisMRennisonJMcGownABromleyMElderRHRaffertyJACrowtherDMargisonGP1993 Expression of *O*^6^-Alkylguanine-DNA alkyltransferase in situ in ovarian and Hodgkin's tumours Eur J Cancer 29A 13061312 8343274 10.1016/0959-8049(93)90079-u

[bib14] MargisonGPO'ConnorPJ1990 Biological consequences of reactions with DNA: role of specific lesionsInHandbook of Experimental Pharmacology 94/1Cooper CS, Grover PL (eds)pp547571Berlin Heidelberg: Springer-Verlag

[bib15] MiddletonMRLunnJMMorrisCRustinGWedgeSRBramptonMHLindMJLeeSMNewellDRBleehenNNewlandsESCalvertAHMargisonGPThatcherN1998 *O*^6^-alkylguanine-DNA alkyltransferase in pretreatment tumour biopsies as a predictor of response to temozolomide in melanoma Br J Cancer 82 11991202 10.1038/bjc.1998.654 PMC2062985 9820180

[bib16] MusarratJWilsonJAAbou-IssaHWaniAA1995 *O*^6^-alkylguanine DNA alkyltransferase activity levels in normal, benign and malignant human female breast Biochem Biophys Res Commun 208 688696 7695624 10.1006/bbrc.1995.1393

[bib17] NCIC, (National Cancer Institute of Canada)2001 Phase II study of temozolomide given in a 7 days on, 7 days off oral schedule to patients with advanced breast cancerhttp://www.ctg.queensu.ca/public/Clinical_Trials/ind_trial_summ.htm#breast

[bib18] PhillipsWPGersonSL1999 Acquired resistance to *O*^6^-benzylguanine plus chloroethylnitrosoureas in human breast cancer Cancer Chemother Pharmaco 44 319326 10.1007/s002800050984 10447580

[bib19] PeggAE1990 Mammalian *O*^6^-Alkylguanine-DNA Alkyltransferase: Regulation and Importance in response to Alkylating Carcinogenic and Therapeutic Agents Cancer Res 50 61196129 2205376

[bib20] PeggAE1994 Repair of *O*^6^-Methylguanine in DNA by Mammalian tissuesInBiochemical Basis of Chemical CarcinogenesisGriem H, Jung R, Kramer M, Marguardt H, Oesch F (eds)pp265274New York: Raven Press

[bib21] PeggAEDolanMEMoschelRC1995 Structure, function and inhibition of *O*^6^-alkylguanine-DNA alkyltransferase Prog Nucleic Acid Res Mol Biol 51 167223 7659775 10.1016/s0079-6603(08)60879-x

[bib22] PetersWPRossMVredenburghJJHusseinARubinPDukelowKCavanaughCBeauvaisRKasprzakS1994 The use of intensive clinic support to permit outpatient autologous bone marrow transplantation for breast cancer Semin Oncol 21Suppl 72531 7916487

[bib23] SpiroTPGersonSLLiuLMajkaSHaagaJHoppelCLIngallsSTPludaJMWillsonJK1999 *O*^6^-benzylguanine: a clinical trial establishing the biochemical modulatory dose in tumor tissue for alkyltransferase-directed DNA repair Cancer Res 59 24022410 10344750

[bib24] Van AgthovenTTimmermansMFoekensADorssersLCJHansen-LogmansSC1995 Differential expression of estrogen, progesterone and epidermal growth factor receptors in normal, benign and malignant human breast tissues using dual staining immunohistochemistry Am J Pathol 144 12381246 PMC1887470 7515559

[bib25] WatsonAJMargisonGP2000 *O*^6^-alkylguanine-DNA alkyltransferase assay Methods Mol Biol 152 4961 10957968 10.1385/1-59259-068-3:49

[bib26] ZaidiNHLiuLGersonSL1996 Quantitative Immunohistochemical Estimates of *O*^6^-alkylguanine-DNA Alkyltransferase Expression in Normal and Malignant Human Colon Clin Cancer Res 2 577584 9816206

